# Parental Attachment and Peer Relationships in Adolescence: A Systematic Review

**DOI:** 10.3390/ijerph19031064

**Published:** 2022-01-18

**Authors:** Elena Delgado, Cristina Serna, Isabel Martínez, Edie Cruise

**Affiliations:** 1 Departament of Psychology, University of Castilla-La Mancha, C/Altagracia 50, 13071 Ciudad Real, Spain; elenadelga97@gmail.com (E.D.); cristina.serna@uclm.es (C.S.); 2 College of Arts and Sciences, Embry-Riddle Aeronautical University, 600 S Clyde Morris Blvd, Daytona Beach, FL 32114, USA; Cruisee@erau.edu

**Keywords:** attachment styles, friendship, peer relationships, adolescence

## Abstract

According to attachment theory, children’s early experiences with their primary caregivers, in terms of protection and security, are the basis for socioemotional development and for the establishment of close relationships throughout their lives. During adolescence, friends and peers become a primary developmental environment, and thereby establishing quality bonds with peers will foster good psychological adjustment. The aim of the present study was to review the evidence on the relation of parental attachment to the quality of peer relationships during adolescence. A systematic review was conducted according to the recommendations of the Preferred Reporting Items for Systematic Reviews and Meta-Analyses guidelines. The search was performed in the PsycInfo, Scopus, and Web of Science (WOS) databases. Inclusion criteria were studies published since 2001, in English, that are academic publications in scientific journals, that explore adolescence, and that analyze the relationship between attachment styles and adolescent peer interactions. The search resulted in 1438 studies, of which 19 studies met the criteria and were included in the review. The results highlighted that secure attachment predicts and promotes the creation of affective relationships with peers and friends based on communication, support, intimacy, trust, and quality. In addition, some variables, such as gender differences or family characteristics, were found to be involved in attachment and provide a better understanding.

## 1. Introduction

Attachment theory is a social-emotional development theory that was originally developed by John Bowlby [1] in order to explain the bond between babies and their caretakers. The basic premise is that an individual’s security and trust toward others in later life stages are molded by their experiences with relationship patterns and the emotional availability of their caretakers, that is to say, their attachment figures. Later, Ainsworth [2] carried out some of the first studies on the individual differences which manifest in attachment, observing how this system is activated and discovering differences based on the behaviors of the caretakers. Through a standardized laboratory procedure called “strange situation”, Ainsworth recorded systematic observations on mother–child interactions in the first year of life, as well as the reaction of the child during separation from and reunion with the mother.

Ainsworth, Blehar, Waters, and Wall [3] proposed the first classification of attachment styles that distinguished between secure, insecure ambivalent, and insecure avoidant attachment. Secure attachment is produced when the caretakers demonstrate physical and emotional warmth, trust, and availability. When placed in the strange situation, in which the attachment figure is not present, the child tends to feel anxious upon being separated from the caretaker and then calm when the caretaker returns [3]. Children with this style of attachment experience comfort with privacy and closeness, tend to search for support, present low anxiety and evasiveness, and confront stress well [4]. Insecure ambivalent attachment occurs when the caretaker is available only on certain occasions. During the strange situation, the child suffers great anguish followed by difficulty calming down when the attachment figure reappears, with fluctuations between anger and worry [3]. Children with insecure ambivalent attachment develop high anxiety, the need for closeness, worry about establishing relationships, and fear of rejection [4]. Lastly, in insecure avoidant attachment, the caretaker does not attend to the baby’s cues that signal the need for protection. In the strange situation under this type of attachment, this child experiences indifference, in addition to anguish and anger in some cases, upon becoming separated from their mother and later demonstrates indifference upon reuniting with her [3]. Children with insecure avoidant attachment develop self-sufficiency and a preference for emotional distancing from others [4]. Years later, Main and Solomon [5] incorporated a fourth category, disorganized attachment. This typology presents characteristics of the two previous styles, insecure ambivalent and insecure avoidant, demonstrating contradictory behaviors and disorganization.

Family has a key function in the development of an individual as the primary group of belonging [6,7]. The first emotional bonds, values, beliefs, and habits are formed within the family [8,9]. Drawing from attachment theory, research established that the emotional and familial history of a person predicts their type of attachment as an adult [10].

### 1.1. Attachment in Adolescence

Attachment theory has expanded in recent decades, with its influence over relationships other than the paternal-filial being explained, as well as how attachment influences in later development stages [11]. In this developmental period, adolescents prepare to develop their potential and begin adulthood [12]. The emotional, cognitive, and social transformations of the adolescent are delineated by attachment processes previously established [13]. Indeed, the empirical evidence indicates that adolescents, in general, experience an increased need for privacy and a decrease in emotional closeness, expressions of affection, and time spent with parents [11]. There are higher levels of ambivalence and lower levels of idealization from adolescents towards their parents [14].

Autonomy development in adolescence entails a continuation of child-like exploration, as well as attachment framework, alongside having to find a balance between new scenarios and needs [15]. Similar to what occurred in the strange situation, adolescents feel more secure when they perceive availability and support from their parents. Therefore, in spite of the paternal-filial relationship changes, when there is a quality relationship, the bond between them is still characterized by warmth, with the parents being important attachment figures even until emerging adulthood [11]. In the process of adolescent development, the internal working models guide the child in the construction of their relational world with their past experiences as the base. The internal working models are frameworks or internal maps of each person in which the relationship with other significant individuals is constructed and represented. According to Bowlby [16], these internal models allow the child to predict and interpret the conduct of their attachment figures given that such internal models define the internal working models consisting of expectations and beliefs about the self and others. In the future, these models are integrated in the personality and guide social relations. “Every situation we meet with in life is construed in terms of the representational models we have of the world about us and of ourselves. Information reaching us through our sense organs is selected and interpreted in terms of those models, its significance for us and those we care for is evaluated in terms of them, and plans of action executed with those models in mind. On how we interpret and evaluate each situation, moreover, turns also how we feel” [17] (p. 229). These models will allow the child or adolescent to evaluate the availability of their attachment figures and act accordingly [18,19].

Individual differences exist in the distancing process of the adolescent with their parents, which could prove more problematic in teens with insecure attachment (insecure-avoidant, insecure-ambivalent, and insecure-disorganized). It has been shown that via secure attachment, conflicts with parents are navigated in a healthy way, with a greater tendency from both parents and child to communicate with each other and find solutions. Adolescents with secure attachment demonstrate confidence defending their opinion to their parents, knowing that there will be no negative consequences and that the relationship will remain intact [13]. However, adolescents with insecure attachment experience emotional distancing from their parents in a stressed manner. These adolescents foresee a threat in the relationship with their parents during the autonomy-seeking process. It has been found that the adolescents with insecure-evasive attachment tend to avoid conflict and opportunities for solutions, while adolescent with insecure-ambivalent attachment show intense involvement which can diminish increasingly throughout the autonomy-seeking process of the adolescent [20].

### 1.2. Adolescence, Friendship, and Attachment

Kerns [21] showed that children generalize the behaviors assimilated with their parents to their peers and friendships that they develop throughout their life so that adolescents with positive parental figures have greater social competency. According to Bowlby [22], the association between attachment and peer relationships can be explained by internal working models (IWM). IWM make it possible for children to know who their attachment figure is and their availability when they need it [23]. Children’s experiences with their caretakers in everyday interactions are integrated to form long-lasting representations with emotional components that will modulate later conduct [24].

Furman, Simon, Shaffer, and Bouchey [25] proved that the IWMs of adolescents with their parents are similar to the IWMs that they establish with their peers and friends. Attachment theorists maintain that the quality of extrafamilial relationships, particularly with peers, is directly influenced by the experiences of attachment with their caretakers [26]. Children with an avoidant attachment style expect rejection in the context of relationships, and therefore they are more likely to be hostile and antisocial with others, inciting others to reject them, such as peers. These behaviors could also be a defense mechanism to protect themselves from others’ rejection. By contrast, children with ambivalent attachment tend to be socially isolated [27]. A secure attachment organization allows for coherency in emotional experiences with peers, while an insecure model is more characterized by exclusion or inability to integrate information, which consequently leads to distorted communication and difficulty in social functioning [13]. Attachment theory suggests that individuals with insecure attachment have a negative image of themselves and others in terms of relationships, resulting in great problems forming intimate bonds in the peer group [28]. Mind theory, the ability to attribute mental states and intentions to others, as well as the ways in which it is related to the quality of attachment present in adolescents has also been studied. It was observed that the sociocognitive constructions and anomalies in the processing of social information is linked to insecure attachment, as well as biased and less positive attributions about their peers [29]. As children move further into adolescence, there is greater integration with the peer group [30]. Friends carry out important functions, with time spent with friends progressively increasing, to the point that self-disclosure and intimacy are reached in some more stable friendships [31]. At this time, conversations where worries related to age garner importance and emotional support is lent [32]. Privacy/intimacy/closeness with friends in adolescence assists in exploration and self-knowledge [33].

On the other hand, classic attachment theory is formulated in sex-neutral terms and does not predict or explain the emergence of differentiated styles according to sex. However, there are findings powerfully challenge the standard sex-neutral model, since many of the outcomes related with individual differences in attachment have differences depending on ecological and social factors [34]. Studies have shown that women and men tend to be socialized differently from birth [35]: men are less emotional and less nurturing than women, and thus they may perceive social relationships differently and, consequently, interact differently. Research is consistent, with the observed gender difference being that when stressed, males tend to engage in “fight or flight” behaviors, while females tend to engage in “tend and befriend” behaviors [36]. Differences by sex in both the structure and the content of peer relationships has been observed [37]. Interactions among boys centers around larger friendship groups with a focus toward comradeship, control, or competence. However, girls are centered more by intimate dyads of friendship based on self-disclosure, emotional expression, and interdependence [38,39]. The scientific literature has suggested that girls, in comparison with boys, have the need to establish more harmonious relationships [39]. Despite the findings in differences by sex, no consensus has been found on the exact nature of these differences in adolescents [40].

### 1.3. Quality of Relationships and Psychological Adjustment

Numerous empirical studies support the importance of friendship and the establishment of bonds in the development of adaptation, especially in childhood and adolescence [41]. For example, Nangle, Erdley, Newman, Mason, and Carpenter [42] showed that friendship predicted short-term psychological well-being. In childhood, the presence of psychological difficulties is associated with peer relationships and the number of close individuals that the child has is related to psychological adjustment and emotional well-being [43]. During childhood and adolescence, friendships provide an environment in which social competencies are developed and self-esteem is formed, boosting health throughout the lifetime. Additionally, peers and friends are a source of instrumental and emotional support, which eases access to other resources, whether they be material or symbolic, which provides for emotional well-being [44].

Adolescents who report having positive relationships present with greater self-esteem and less anxiety or depression. Some authors affirm that interpersonal loss or not forming close, supportive relationships contributes to clinical symptomology [45]. For the majority of people, relationships with others forms a central point in their lives. The development, maintenance, and dissolution of bonds are sources of intense emotions, both joy and happiness as well as angst and sadness. Individuals who have positive and long-lasting relationships have lower mortality rates, less depression, and a lower presence of psychological and physical health problems. Contrastingly, people with weak links have greater rates of mortality, loneliness, unhappiness, and depression [46].

In attachment research, it has been demonstrated that adolescents who have insecure attachment representations tend to be more hostile and anxious with their peers than adolescents with secure attachment representations [47]. Similarly, self-concept also plays an important role in social competency. Adolescents who are considered to be well-regarded by their friends score higher in self-esteem. On the other hand, considering oneself as lacking in social ability has a negative influence on self-esteem, which can even lead to depressive feelings [45,48]. As Campbell [49] indicated, dissatisfaction with oneself produces a more damaging effect on the feeling of well-being than dissatisfaction with any other domain of life. A study by Cole, Martin, Powers, and Truglio [50] showed that adolescents’ perception of their relational competency predicted depression months later; thus, perceiving oneself as lacking in this ability is a risk factor for mental health. Therefore, the extent to which adolescents establish and maintain quality, positive relationships with their peers is considered a protective factor in social-emotional adjustment throughout the lifetime, which supports better adaptation [51].

### 1.4. Previous Studies

Bowlby’s attachment theory on the emotional bond of a child with their caretakers was formed decades ago, and therefore it has changed and evolved, being adapted to new times. Researchers have studied and considered the influence of other variables, extending the concept to other ages and relationships beyond baby–attachment figure [52]. Some characteristics of today’s society are different from those at the time Bowlby’s theory was developed [1], such as the increase in single parenthood, partner relationships, the temperament of the baby, or social support outside of the family. Additionally, studies have gone beyond childhood to include the full lifetime [53]. Due to this, efforts have been made to keep the theory up to date, incorporating advances made in psychology, which is one of the reasons why the theory has maintained its relevance throughout the years. This is due to the need for greater theoretical clarity on the repercussions of attachment in future behavior, which is one of the challenges of attachment theory studies in the 21st century [54].

What has been studied previously in some reviews is how attachment formed with parents at the start of life influences in establishing bonds with peers in later years, such as in the review by Sheneider, Atkinson, and Tardif [55], or in the meta-analysis carried out by Pallini, Baiocco, Schneider, Madigan, and Atkinson [56]. However, these studies focused on the childhood stage or in childhood combined with adolescence. Some publications indicate that during childhood and adolescence, children with secure attachment show greater social competence and have more positive relationships with their peers and friends compared to children with insecure attachment [51]. The present review focuses on the relation between attachment and relationships with peers in adolescence given that good adjustment in an adolescent’s social relationships determines, to a large degree, adult life, boosting psychological well-being and decreasing the risk of psychopathologies. The review focuses on adolescence, which is a complex stage characterized by change and the transition from childhood toward adult life. The World Health Organization (WHO) defines adolescents as those people between 10 and 19 years of age [57].

The main objective of this work is to analyze the adolescents’ bond with their peers by attachment type established in childhood with their main caretakers, considering differences between girls and boys. Peer bond is analyzed by quality, privacy/closeness/intimacy, and reciprocity of the relationship.

## 2. Materials and Methods

The systematic review was conducted according to the Preferred Reporting Items for Systematic Reviews and Meta-Analyses (PRISMA) guidelines, adhering to the PRISMA 27-item checklist [58].

### 2.1. Search Strategy

The search of the sources was carried out on PsycInfo, Scopus, and Web of Science (WOS) databases, as they collect studies most prominent in the field of psychology. We used these databases to identify articles that were published between 1 January 2001 and 31 December 2020. We chose to limit the search in order to analyze articles published in the 21st century until the date of the search. It should be taken into account that the 21st century coincides with the rise of the study of attachment [52]. The search terms used as keywords were attachment, peer relation, friendship, adolescence, and adolescent; they were entered in combinations of 3 blocks of terms applying the Boolean operators “and” and “or”.

### 2.2. Inclusion and Exclusion Criteria

For inclusion in this study, the studies had to meet the following criteria: (1) studies published since 2001 in the PsycInfo, Scopus, or Web of Science databases; (2) studies published in English; (3) academic publications in scientific journals; (4) studies that explore the adolescent population, following WHO criteria (10–19 years old); and (5) studies that analyze the relation between attachment styles and adolescent interactions with their peers.

For exclusion in this study, the studies had to meet the following criteria: (1) studies published prior to 2001 in the PsycInfo, Scopus, or Web of Science databases; (2) studies in languages other than English; (3) documents that are not academic publications in scientific articles, such as book chapters or dissertations; (4) studies that explore attachment relationships in child or adulthood; and (5) studies that do not analyze the relations between attachment styles and adolescent interactions with their peers.

## 3. Results

### 3.1. Selection of Studies

**Figure 1:**
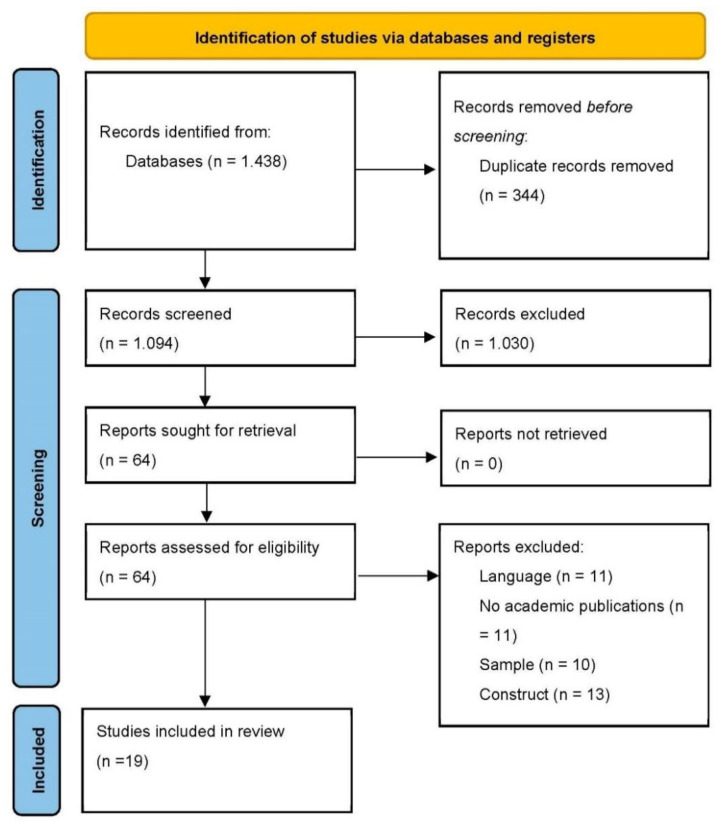
PRISMA flow chart of the study selection process.

A total of 1438 articles were identified with the initial chosen search terms. After removal of duplicates, a total of 1094 publications were considered for the analysis. After reading the title and synopsis, we excluded 1030 articles, leaving 64 full-text articles that were assessed for eligibility. Of the remaining publications, 45 studies were excluded due to not meeting the inclusion criteria. We used Cohen’s kappa to calculate the inter-coder agreement between the two authors. The inter-coder agreement was 95.31% (Cohen’s k = 0.89), almost perfect agreement. The few discrepancies between the coders were solved through discussion. Figure 1 shows the flowchart of the review process.

### 3.2. Descriptive Characteristics of the Included Studies

Descriptive details of the eligible studies are presented and summarized in Table 1 and Table 2. The articles selected were published in the 21st century, between 2001 and 2020. We can observe that the country that carried out the most research on the subject analyzed was the United States (*n* = 11), followed by Israel (*n* = 2), Spain (*n* = 2), and Canada (*n* = 2), with the lowest production in Germany and the United Kingdom. Regarding the study design, studies have followed predominantly a cross-sectional design (*n* = 15), and only four studies presented a longitudinal design that used either three (*n* = 2), four (*n* = 1), or five (*n* = 1) time points. In reference to the characteristics of the sample, all studies used adolescent participants, with a wide variety in terms of the adolescent stage. The mean age of participants across studies ranged from 11.9 to 18.38 years, and one included university students. Eighteen studies included the topic among samples balanced for gender, and only one study [59] had an exclusively male sample.

### 3.3. Measures

Attachment was assessed using a wide variety of measures, a total of 12. Different forms of the Adult Attachment Interview (AAI) [60] were the most common measure of assessing attachment, used in 7 studies, since modified versions were used (adapted to the age of the participants) or derivation such as the Q-SORT or Q-SET. Other measures that were recurring in various studies were Inventory of Parent and Peer Attachment (IPPA) [61], *n* = 2; The Adult Attachment Measure [62], *n* = 2; and Relationship Questionnaire (RQ) [63], *n* = 2. The study by Allen et al. [64] analyzed autonomy and relatedness, designing their own instrument via observation and codification of the behaviors in the sample.

There was a heterogeneity of the instruments for the peer relation and friendship used, with a total of 27. The questionnaires most adopted were the Perceived Competence Scale for Children [65], Network of Relationships Inventory (NRI) [66], Friendship Qualities Scale (FQS) [67], Intimacy Scale [68], and Adolescent Self-Perception Profile [69]. The study by Weimer et al. [70] analyzed friendship interaction processes, designing their own instrument via observation and codification of the behaviors in the sample. Some studies included in the review used a wider focus, taking into account other relevant aspects that can affect the peer interaction process.

The mediators explored in the reviewed literature were the following: Self-Esteem Scale [71], Depressive Mood List [72], Marital Relationship [73], Offer Self-Image Questionnaire [74], Child Depression Inventory [75], Child Behavior Checklist [76], Movie for the Assessment of Social Cognition (MASC) [77], and International Personality Inventory Pool [78].

### 3.4. Attachment Styles and Relation with Peers

The studies reviewed show the implications of attachment styles on the interaction of adolescents with peers. Adolescents in the studies who exhibited secure attachment developed in an environment dominated by parental warmth, autonomy, and resolving capacity. It has been observed that these adolescents have integrated positive interaction models, acquiring competencies that allow them to establish bonds of friendship based on intimacy and closeness, fluid communication, and comfort exploring and interacting with friends [59,64,79,80,81,82,83,84].

In adolescence, there is an increase in intimacy among peers, as friends become the people of reference. Adolescents with secure attachment develop intimacy, which allows them to share ideas and feelings safely. They acquire social skills that allow them to maintain fluid conversations based on assertiveness, with the ability to overcome the difficulties that may arise in the interaction [46,51,70,85,86]. In addition, they are more receptive to seek and receive support from friends in moments of need [87].

This is the opposite in adolescents with insecure attachment. They show lower competencies to establish bonds with their peers, such as difficulty in creating friendships based on intimacy and communication. Adolescents with ambivalent attachment representations show low competence to regulate conflicts with a best friend cooperatively and show high scores in hostility and social anxiety. Adolescents with anxious attachment experience intense reactions, both positive and negative, in their close relationships. This behavioral pattern involves high levels of negativity in relation to negative peer behavior, as well as high levels of positivity in relation to peer acceptance, while adolescents with avoidant attachment representations do not value close relationships and describe themselves as emotionally independent, alongside not feeling the need to share ideas or feelings with others. Adolescents with avoidant attachment worry about getting hurt if they trust others. They have low expectations about what friendship means (communication, trust, intimacy, or emotional support) [87,88,89,90,91].

### 3.5. Sex Differences

The literature suggests that differences by sex exist. Seven of the selected articles explored these differences, and of these, 100% reported a positive relation. Girls showed greater sociability and closer interactions in the relationships they established with their peers. These relationships are based on affective bonds characterized by closeness and intimacy. For girls, friendship is a space where they can be supported and receive comfort. However, boys show a tendency to have less affective relationships. The relationships they establish are more focused on sharing time or doing activities with their friends [79,83,88,89,92]. In addition, among girls, it is more frequent to establish a secure bond, with greater warmth and affection, while with boys it is more frequent to establish a colder bond with less affection [81].

Another finding is that securely attached adolescents interact comfortably with same-sex and opposite-sex friends. However, those adolescents with insecure attachment, both avoidant and anxious, show a tendency to have interactions with same-sex and less with opposite-sex friends [85]. 

**Table 1:** Descriptive characteristics of included studies.

Author/Year	Country	Type of Research		Sample	
			N	Age	Gender (Males)
Engels et al., 2001 [46]	USA	Cross-sectional	412	Early adolescents (mean = 13.0 SD = 0.82) Middle adolescents (mean = 16.5 SD = 1.09)	Not reported
Mikulincer and Selinger, 2001 [79]	Israel	Cross-sectional	193	15–16 (mean/SD = not reported)	93 (48.20%)
Markiewicz et al., 2001 [80]	Canada	Cross-sectional	69	16–12 (mean/SD = not reported)	24 (34.80%)
Sánchez-Queija and Oliva, 2003 [81]	Spain	Cross-sectional	513	13–19 (mean = 15.4 SD = 1.19)	221 (43.10%)
Weimer et al., 2004 [70]	USA	Cross-sectional	44 pairs (88)	15–18 (mean = 16.3 SD = not reported)	34 (38.60%)
Zimmermann, 2004 [82]	Germany	Cross-sectional	43	16 (mean/SD = not reported)	22 (51.20%)
Saferstein et al., 2005 [85]	USA	Cross-sectional	330	17–22	112 (33.90%)
Allen et al., 2007 [64]	USA	Longitudinal	167	13–16 Wave 1 (mean = 13.4)	80 (47.90%)
				Wave 2 (mean = 14.3) Wave 3 (mean = 15.2)	
Dykas et al., 2008 [51]	USA	Cross-sectional	189	16–17 (mean = 17/ SD = not reported)	71 (37.60%)
Feeney et al., 2008 [87]	USA	Cross-sectional	135	15–18 (mean = 16.5 SD = 0.58)	51 (38%)
Bauminger et al., 2008 [88]	Israel	Cross-sectional	196	15–12 (mean/SD = not reported)	116 (59.20%)
Shomaker and Furman, 2009 [89]	USA	Cross-sectional	200	14–16 (mean = 15.3)	100 (50%)
Carr, 2009 [59]	UK	Cross-sectional	96	mean = 13.1 SD = 1.01	96 (100%)
Boling et al., 2011 [86]	USA	Cross-sectional	113	14–12 (mean = 12.7 SD = 0.7)	51 (45.10%)
Sánchez-Queija and Oliva, 2015 [83]	Spain	Longitudinal	101	W1 (mean = 13.1) W2 (mean = 15.4) W3 (mean = 17.8)	38 (37.60%)
Venta et al., 2015 [90]	USA	Cross-sectional	271	17–12 (mean = 15.95 SD = 1.43)	103 (38%)
Chow et al., 2016 [91]	USA	Longitudinal	223	18–11 W1 (mean = 11.90	115 (51.60%)
				SD = 0.43) W2 (mean = 14.20 SD = 0.46) W3 (mean = 16.17 SD = 0.44) W4 (mean = 17.84 SD = 0.46)	
Wong et al., 2020 [92]	Canada	Cross-sectional	776	13–19 (mean = 15.2 SD = 1.52)	13–19 (mean = 15.2 SD = 1.52)
Loeb et al., 2020 [84]	USA	Longitudinal	184	14–18 W1 (mean = 14.27 SD = 0.77) W2 (mean = 15.21 SD = 0.81) W3 (mean = 16.35 SD = 0.87) W4 (mean = 17.32 SD = 0.88) W5 (mean = 18.38 SD = 104)	86 (46%)

**Table 2:** Descriptive characteristics of included studies.

Author/Year	Measures			Results	
	Attachment	Relationships	Other	Attachment Styles and Relation with Peers	Gender Differences
Engels et al., 2001 [46]	- Inventory of Parent and Peer Attachment (IPPA, Armsden and Greenberg, 1987 [61]) - Adolescent version of the Interpersonal Behavior (SIG; Arrindell, De Groot, and Walburg, 1984 [93]; Bijstra, Jackson, and Bosma, 1995 [94])	- Perceived Competence Scale for Children (Harter, 1985 [65])	- Self-Esteem Scale (Rosenberg, 1965 [71]) - Depressive Mood List (Kandel and Davies, 1982 [72])	Higher parental attachment predicts development of adolescents’ interpersonal skills such as competencies in initiating and maintaining, criticizing, or being assertive.	Not reported
Mikulincer et al., 2001 [79]	- Descriptions of attachment styles (Hazan and Shaver, 1987 [62])	- Acquaintance Description Form (ADF-F; Wright, 1984, 1985 [73,95]) - Adaptation version Network of Relationships Inventory (NRI; Furman and Buhrmester, 1985 [66])	-	Adolescents with secure attachment give greater importance to closeness, support, and affiliation with their friends and peers.	YES
Markiewicz et al., 2001 [80]	- Adaptation version of the Relationship Questionnaire (RQ; Bartholomew and Horowitz, 1991 [63]).	- Adolescents’ prosocial behavior scale was taken from Feelings and Behavior Questionnaire (Statistics Canada, 1995 [96]) - Friendship qualities scale (FQS; Bukowski et al., 1994 [67])	- Perceptions of the Marital Relationship scale from the Spanier Dyadic Adjustment Scale (Wright, 1985 [73]) - The Perception of Mother’s Social Network scale was constructed from the Social Relationship Network Questionnaire (Veroff, 1996 [97])	Adolescents with secure attachment present better quality in their relationships with friends.	Not reported
Sánchez-Queija and Oliva, 2003 [81]	- Parental Bonding Instrument (Parker, Tupling and Brown, 1979 [98])	- Inventory of Parent and Peer Attachment (IPPA, Armsden and Greenberg, 1987 [61]) - Intimacy Scale (Sharabany, 1994 [68])	-	Adolescents with secure attachment present better affective relations with their friends, characterized by closeness and identity.	YES
Weimer et al., 2004 [70]	- Relationship Questionnaire (Bartholomew and Horowitz, 1991 [63])	- Observation and coding of friendship interaction processes - Coding categories: High and low self-disclosure, gossip, problem solving, connectedness, individuality, transactive statements, planning, and extraneous comments - Friendship Qualities Scale (FQS; Bukowski, Hoza, and Boivin, 1994 [67])	-	Adolescents with secure attachment establish relationships with friends based on intimacy, connection, and fluidity.	Not reported
Zimmermann, 2004 [82]	- AAI Q-sort (Kobak, 1993 [99])	- Friendship and Peer Relations Interview (Zimmermann, 1992 [100])	- Offer Self-Image Questionnaire (Seiffge-Krenke, 1987 [74])	Adolescents with secure attachment present a more elaborate concept of friendship, better quality relations, and greater intimacy with peers.	Not reported
Saferstein et al., 2005 [85]	- Adult Attachment Measure (Hazan and Shaver, 1987 [62])	- Friendship Qualities Scale (FQS; Bukowski et al., 1994 [67])	-	Adolescents with secure attachment report greater quality in their interactions, comradeship, transcendence in the problems that arise in interactions, and les conflict with peers.	YES
Allen et al., 2007 [64]	- Adaptation version of the Adult Attachment Interview (AAI) and Q-set (Kobak et al., 1993 [101]) - Observed autonomy and relatedness with parent - Coding categories: promoting relatedness, undermining relatedness, promoting autonomy, and undermining autonomy	- A modified version of the Conflict Tactics Scale (Straus, 1979 [102]) - Supportive Behavior Coding System (Allen, Hall, Insabella, Land, Marsh and Porter, 2001 [103]) - The Autonomy-Relatedness Coding System for Peer Interaction (Allen, Porter, and McFarland, 2001 [104]). - Measure of popularity following the procedure described in Coie, Dodge, and Coppotelli (1982) [105] - Inventory of Parent and Peer Attachment (Armsden and Greenberg, 1987 [61])	- Child Depression Inventory (Kovacs and Beck, 1977 [75]) - Child Behavior Checklist (Achenbach and Edelbrock, 1991 [76])	Secure attachment and positive tone with parents in disagreements is linked to positive relations based on emotional support, popularity, and les pressure with peers.	Not reported
Dykas et al., 2008 [51]	- Modified version of AAI (George et al., 1985 [60]).	- Modified version of social behavior and victimization assessments developed by Parkhurst and Asher (1992) [106] - Peer acceptance assessment using an instrument devised by Asher and Dodge (1986) [107] - Nomination procedure A modified version of the Children’s Expectations of Social Behavior Questionnaire–Peer Version (Rudolph, Hammen, and Burge, 1995 [108])	-	Adolescents with secure attachment are perceived as more prosocial and are more accepted by peers.	Not reported
Feeney et al., 2008 [87]	- Adult Attachment Interview (AAI; George, Kaplan, and Main, 1985 [60])	- Modified version of scale support-seeking and support provision by Collins and Feeney, 2000 [109], and Feeney, 2004 [110] - Experiences in Close Relationships Scale (ECR; Brennan, Clark, and Shaver, 1998 [111])	-	Adolescents with secure attachment representations present greater support-seeking and support-giving behaviors with strangers of similar age.	Not reported
Bauminger et al., 2008 [88]	- Attachment Styles Questionnaire (ASQ; Mikulincer, Florian, and Tolmacz, 1990 [112])	- Intimacy Scale (Shulman, Laursen, Kalman, and Karpovsky, 1997 [113]) - Adolescent Sense of Coherence Scale (Margalit and Ziv, 1997 [114]) - Self-Disclosure Scale (Shulman et al., 1997 [113])	-	Avoidant and anxious attachment are related to less intimacy with peers, mediated by low self-coherence and self-disclosure.	YES
Shomaker et al., 2009 [89]	- Network of Relationships Inventory (NRI): Behavioral Systems Version (Furman, 2000 [115]) - Adult Attachment Interview (AAI; George, Kaplan, and Main, 1985 [60]) - Behavioral Systems Questionnaire (BSQ; Furman and Wehner, 1999 [116])	- Adolescent–close friend dyads. - Using Interactional Dimensions Coding System (IDCS; Julien, Markman and Van Widenfelt, 1986 [117])	-	Dismissing working models are associated with poorer focus on problem discussions and weaker communication skills.	YES
Carr, 2009 [59]	- Adolescent Attachment Questionnaire (AAQ; West, Rose, Spreng, eldon-Keller and Adam, 1998 [118])	- Sport Friendship Quality Scale (SFQS; Weiss and Smith, 1999 [119])	-	Adolescent dyads with secure attachment show characteristics of more positive friendship than those with one member with insecure attachment.	Not reported
Boling et al., 2011 [86]	- Parental Attachment Questionnaire (PAQ; Kenny, Moilanen, Lomax, and Brabeck, 1993 [120])	- Self-perception Profile for Adolescents (Harter, 1988 [69]) - Friendship Qualities Questionnaire - (FQQ; Berndt and Keefe, 1995 [121])	-	Secure attachment is related with adolescent social competence and higher quality in friendship with peers. Adolescents with secure attachment feel comfortable exploring their environment and interacting.	Not reported
Sánchez-Queija and Oliva, 2015 [83]	- Parental Bonding Instrument (PBI; Parker, Tupling, and Brown, 1979 [98])	- Intimacy Scale (Sharabany, 1994 [68]) - Peer-group Attachment Scale (Armsden and Greenberg, 1987 [61])	-	Adolescents with secure attachment present greater closeness and intimacy toward their peers and best friends, demonstrating a similar tendency across ages.	YES
Venta et al., 2015 [90]	- Child Attachment Interview (CAI; Target, Fonagy, Shmueli-Goetz, Datta, and Schneider, 2007 [122])	- Social Problems subscale of the Child Behavior Checklist (CBCL; Achenbach and Rescorla, 2001 [123])	- Movie for the Assessment of Social Cognition (MASC; Dziobek, Fleck, Kalbe, Rogers, Hassenstab, Brand, …, and Convit, 2006 [77])	Adolescents with disorganized attachment have difficulty in their interpersonal relations with peers, with mentalizing mediating	Not reported
Chow et al., 2016 [91]	- A modified version of the Adult Attachment Scale (AAS; Collins and Read, 1990 [124])	- Network of Relationships Inventory (Furman and Buhrmester, 1985 [66])	-	Avoidant attachment is associated with past perceptions of exclusion in friendship and a decrease in intimacy.	Not reported
Wong et al., 2020 [92]	- Comprehensive Adolescent-Parent Attachment Inventory (CAPAI; Moretti, McKay, and Holland, 2000 [125])	- Network of Relationships Inventory-Social-Provision Version (NRI-SPV; Furman and Buhrmester, 1985 [66])	-	Adolescents with anxious attachment are more likely to establish negative interactions with friends. The older they are, the worse their relations.	YES
Loeb et al., 2020 [84]	- The Adult Attachment Interview Q-Set (AAI Q-Set; Kobak, 1993 [101])	- Supportive Behavior Task (using the Supportive Behavior Coding System) (Allen et al., 2001 [103]) - Aggressive Attitudes Questionnaire (Slaby and Guerra, 1988 [126]) - Adolescent Self-Perception Profile (Harter, 1988 [69])	- International Personality Inventory Pool (Goldberg, Johnson, Eber, Hogan, Ashton, Cloninger, and Gough; 2006 [78])	Adolescents with secure attachment demonstrate more support-seeking behaviors with peers, developing positive relations in later stages, whereas ambivalent insecure attachment is associated with decreased support-seeking.	Not reported

## 4. Discussion

Bowlby’s attachment theory on the emotional bond of a child with their caretakers was formed in the 1960s [1]. Since then, research has incorporated new findings that allow for a greater understanding of the topic, such as the work of Ainsworth [3], in which various types of attachment are differentiated. Later, new variables were incorporated, such as the importance of the role of the parent, the quality of the marital relationship, the increase in single parenthood, social support outside of the family, or the extension of concepts to other life stages such as adolescence or adulthood. The study of attachment theory repercussions, originally analyzed in childhood, has been expanded to other ages, such as adolescence and adult life. This revision centers on the implications that attachment established in childhood has on adolescents later with their peers. The findings of the review carried out on the relation between attachment and adolescent peer interaction are congruent with Bowlby’s theory [1,16,17,22], supporting the hypothesis that secure attachment is positively associated with quality relations between adolescents and their peers. It can be observed that adolescents with secure attachment demonstrate positive interactions based on emotional support and fluid communication. These adolescents report ease seeking and giving support, as well as finding a space among their peers where they feel safe. Therefore, adolescents with secure attachment would extrapolate the pattern of behavior they learn in childhood toward these peer relations with their attachment models.

The results extracted from some studies review, such as Allen et al. [64], Shomaker et al. [89], and Zimmermann [82], show that adolescents who have secure attachment as a base integrate their past experiences favorably, present a more developed concept of friendship, have closer emotional friendships, are more greatly integrated in their peer group, and have a greater emotional regulation ability. Hence, adolescents with secure attachment incorporate internal working models with which they learn patterns of interaction, which in turn promote quality relations with peers and friends. In this way, positively navigating discussions with parents serves as a model so that adolescents later have an ability to resolve conflicts with their peers effectively. These results are in line with Bowlby’s [16] ideas on the continuity of the attachment relationship established in childhood with main caretakers and the relationships developed in later ages. The study by Boling et al. [86] suggests an indirect pathway between parent–adolescent attachment and quality of friendships by way of social competence.

One characteristic of friendship relations is that they are based on intimacy, especially in adolescence, with a distancing from the family environment occurring while friends being to be the main stage where new needs are developed. The results of the review carried out support this idea, showing that the intimacy that adolescents form with peers and friends is mediated by attachment figures, whereby adolescents with secure attachment develop greater intimacy with their peers [70,79,82,83]. On the contrary, when insecure, avoidant, or ambivalent attachment is established, the relations maintained are less intimate and characterized by more difficulty to generate an environment in which competencies are developed, which, in turn, allow adolescents to share ideas or feelings with others. The importance that intimacy has in the development of adolescent autonomy is confirmed since, upon sharing ideas with others, adolescents feel secure and do not fear losing their identity [31]. Adolescents with ambivalent attachment score high in hostility and anxiety, while also showing difficulties managing conflict with friends in a cooperative way. These adolescents experience intense reactions in their close relationships, with high levels of negativity when facing peer rejection and high levels of positivity when faced with peer acceptance. In contrast, adolescents with avoidant attachment do not value close relationships and perceive themselves as emotionally independent. They tend to avoid emotional engagement, demonstrating coldness and distance with their peers, having low expectations about what friendship means. Some authors place the cause of these behaviors within their fear of suffering emotional harm [70,79,82,83].

Regarding social abilities, adolescents with secure attachment possess social abilities and competencies that allow them to explore relations and interact with strangers of the same age in a comfortable manner while feeling secure. Such abilities provide them with assertiveness and the ability to realize feedback in a positive way. On the contrary, the lack of social abilities can be a relational barrier in cases where attachment representations were not secure. When an infant grows in an environment that provides them an insecure environment, in adolescence, the individual will present negative and hostile expressions of affect, as well as violent conduct toward their peers, and will also be less accepted by their peers [46,51,87]. Additionally, according to Loeb et al. [84], opposite to adolescents with secure attachment, those with insecure attachment are less likely to ask for and receive support from their peers; they close themselves off, becoming overly self-sufficient and the likelihood to develop negative relations in later life stages increases. For these authors [84], peer support is a central mechanism that maintains the continuity of the attachment model constructed early on. Relations with peers becomes a productive context in which to practice or reinforce security seeking dynamics developed in childhood.

With respect to the influence of sex, the results show differences between boys and girls. In general, it has been observed that girls maintain conduct patterns characterized by greater sociability, company, protection, and intimacy, establishing relations oriented more toward emotional support. On the other hand, boys tend to relate more instrumentally through shared activities. In addition, boys tend to experience greater relation and security with peers of the same sex rather than the opposite, whereas this does not occur with girls [79,83,88,89,92]. Among girls, it is more common to establish a secure bond, with greater warmth and affection, while with boys it is more common to establish a colder bond with less affection. These differences could be related to cultural values and gender roles developed in childhood [81,127]. In terms of influence of attachment type in adolescent relations, it can be seen that adolescents with secure attachment tend to relate indistinctly with friends of the opposite and same sex. However, adolescents with insecure attachment present greater ease of interaction with peers of the same sex. Numerous authors suggest that maintaining friendships with peers of the opposite sex could entail a source of stress upon perceiving an affective difference. Hence, adolescents with ambivalent attachment tend to worry while adolescents with avoidant attachment are inclined toward minimization or indifference [85].

Moreover, other variables have an influence on the interaction of adolescents with their peers. For example, it has been found that when the quality of the marital relationship between parents is good and the adolescent perceives it as such, there is a greater likelihood for secure attachment to develop and, therefore, greater closeness with friends [80]. On the other hand, it is important for infants to be able to establish a securely attached bond with at least one of the parents in order for the child to acquire the necessary competencies to explore and self-regulate in the future [81]. Another essential aspect is the type of attachment that peers present when relating to the adolescent. Studies have found that dyads in which both friends present secure attachment experience friendship in a more positive way, with connection, support, and fluid conversations, compared to those in which one of the members has insecure attachment [59,70]. Finally, despite few studies existing that examine the disorganized attachment population, Venta and Sharp [90] highlight that attachment influences in mentalizing and in information processing. They observed that subjects with disorganized attachment made more errors in such processing due to difficulty considering the mental states of others. Adolescents with disorganized attachment tend to have problems with peers that interfere in their interpersonal functioning, the opposite of what occurs with those who have experienced secure attachment.

The present review does present some limitations. Firstly, some of the studies analyzed do not clearly differentiate between the distinct types of insecure attachment and are limited to the mere presence or lack of security. Additionally, the number of studies that consider disorganized attachment is limited. Future studies should contemplate the different types of insecure attachment and address disorganized attachment. Given the current predominance of interactions via the internet among the adolescent population, analyzing the differences between online friendship relations and offline friendship relations according to attachment style would also be an important aspect to analyze in future studies. Ultimately, the present review highlights that secure attachment in childhood is related to quality bonds with peers in adolescence.

## 5. Conclusions

The systematic review carried out suggests that the relations which adolescents establish with their peers are directly influenced by the attachment models developed in the first years of life with their main caretakers, and that such models are carried throughout later years. Specifically, secure attachment predicts and fosters relations based on intimacy, trust, good communication, integration, emotional support, and quality relations with friends and peer groups. In addition, the results show other factors to consider in the relationship between secure attachment and posterior adolescent interactions with peers. These factors include family characteristics, mentalizing, and sex.

Therefore, the development of a securely attached base allows for the creation of necessary competencies to maintain social interactions based on affection in adolescence. Adolescents with secure attachment will also be more highly accepted by their peer group, have ease creating and maintaining positive and quality relations with friends and peers. Similarly, secure attachment provides patterns of behaviors adapted to the context of peers, given that adolescents with insecure attachment tend to demonstrate more hostility and aggression. Additionally, some differences by sex were found in interactions, and it is accepted that girls and boys are socialized differently and perceive and behave differently in their relationships. Girls demonstrate greater sociability and emotional expression, finding a space for intimacy in the friend group where self-disclosure can occur and, in turn, development can be supported. Boys’ friendships tend to be based on enjoyment and comradeship through carrying out shared activities. Lastly, adolescents with secure attachment tend to relate indistinctly with friends of the opposite as well as the same sex.

Finally, the impact that promoting security and the support that attachment figures lend in terms of adolescent psychological adjustment must be emphasized. In adolescence, friends become the main relational environment and an important source of support, significantly influencing self-esteem and emotional well-being of the adolescent Having a securely attached base provides social abilities and adaptational capacities with peers, thereby also strengthening the adolescent’s psychological adjustment.

## Data Availability

The study did not report any data.

## References

[1] Bowlby J. (1969). Attachment and Loss, Volume 1: Attachment.

[2] Ainsworth M.S. (1989). Attachments beyond infancy. Am. Psychol..

[3] Ainsworth M.D.S., Blehar M., Waters E., Wall S. (1978). Patterns of Attachment: A Psychological Study of the Strange Situation.

[4] Mikulincer M., Shaver P.R., Pereg D. (2003). Attachment theory and affect regulation: The dynamics, development, and cognitive consequences of attachment-related strategies. Motiv. Emot..

[5] Main M., Solomon J., Brazelton T.B., Yogman M.W. (1986). Discovery of an insecure-disorganized/disoriented attachment pattern. Affective Development in Infancy.

[6] Serna C., Martínez I. (2019). Parental Involvement as a Protective Factor in School Adjustment among Retained and Promoted Secondary Students. Sustainability.

[7] Martínez I., Murgui S., Garcia O.F., Garcia F. (2021). Parenting and Adolescent Adjustment: The Mediational Role of Family Self-Esteem. J. Child Fam. Stud..

[8] Galdós J.sS., Sánchez I.M. (2010). Relationship between cocaine dependence treatment and personal values of openness to change and conservation. Adicciones.

[9] Saiz J., Álvaro J.L., Martínez I. (2011). Relation between personality traits and personal values in cocaine-dependent patients. Adicciones.

[10] Dávila Y. (2015). La influencia de la familia en el desarrollo del apego. An. Univ. Cuenca..

[11] Oliva A.D. (2011). Apego en la adolescencia. Acción Psicol..

[12] Borrás-Santisteban T. (2014). Adolescencia: Definición, vulnerabilidad y oportunidad. Correo Cient. Méd..

[13] Allen J.P., Cassidy J., Shaver P.R. (2008). The attachment system in adolescence. Handbook of Attachment: Theory, Research, and Clinical Applications.

[14] Collins W.A., Steinberg L., Eisenberg N., Damon W., Lerner R.M. (2006). Adolescent development in interpersonal context. Handbook of Child Psychology: Social, Emotional, and Personality Development.

[15] Delgado I.G., Oliva A.D., Sánchez-Queija I. (2011). Apego a los iguales durante la adolescencia y la adultez emergente. An. Psicol..

[16] Bowlby J. (1979). The Making and Breaking of Affectional Bonds.

[17] Bowlby J. (1980). Attachment and Loss, Volume 3: Loss, Sadness and Depression.

[18] Cook W.L. (2000). Understanding attachment security in family context. J. Pers. Soc. Psychol..

[19] Duchesne S., Larose S. (2007). Adolescent parental attachment and academic motivation and performance in early adolescence. J. Appl. Soc. Psychol..

[20] Allen J.P., Land D., Cassidy J., Shaver P.R. (1999). Attachment in adolescence. Handbook of Attachment: Theory, Research, and Clinical Applications.

[21] Kerns K.A., Bukowski W.M., Newcomb A.F., Hartup W.W. (1996). Individual differences in friendship quality: Links to child-mother attachment. The Company They Keep: Friendship in Childhood and Adolescence.

[22] Bowlby J. (1980). Attachment and Loss, Volume 2: Separation.

[23] Marrone M., Diamond N., Juri L. (2001). La Teoría Del Apego: Un Enfoque Actual.

[24] Pinedo P.P., Santelices A.A.P. (2006). Apego adulto: Los modelos operantes internos y la teoría de la mente. Ter. Psicol..

[25] Furman W., Simon V.A., Shaffer L., Bouchey H.A. (2002). Adolescents’ working models and styles for relationships with parents, friends, and romantic partners. Child Dev..

[26] Thompson R.A., Eisenberg N., Damon W., Lerner R.M. (2006). The Development of the Person: Social Understanding, Relationships, Conscience, Self. Handbook of Child Psychology: Social, Emotional, and Personality Development.

[27] Rosenblum K.L., Dayton C.J., Muzik M., Zeanah C.H. (2009). Infant social and emotional development: Emerging competence in a relational context. Handbook of Infant Mental Health.

[28] Bartholomew K. (1990). Avoidance of intimacy: An attachment perspective. J. Soc. Pers. Relatsh..

[29] Dykas M.J., Cassidy J. (2011). Attachment and the processing of social information across the life span: Theory and evidence. Psychol. Bull..

[30] Poncela A.M.F. (2014). Adolescencia, crecimiento emocional, proceso familiar y expresiones humorísticas. Educar.

[31] Cassidy J. (2001). Truth, lies, and intimacy: An attachment perspective. Attach. Hum. Dev..

[32] Spangler G., Zimmermann P. (2014). Emotional and adrenocortical regulation in early adolescence: Prediction by attachment security and disorganization in infancy. Int. J. Behav. Dev..

[33] Parker J.G., Gottman J.M., Berndt T.J., Ladd G.W. (1989). Social and emotional development in a relational context: Friendship interaction from early childhood to adolescence. Peer Relationships in Child Development.

[34] Del Giudice M. (2019). Sex differences in attachment styles. Curr. Opin. Psychol..

[35] Bem S.L. (1993). The Lenses of Gender: Transforming the Debate on Sexual Inequiality.

[36] Scharfe E. (2016). Sex differences in attachment. Encyclopedia if Evolutionary Psychological Science.

[37] De Goede I.H., Branje S.J., Meeus W.H. (2009). Developmental changes and gender differences in adolescents’ perceptions of friendships. J. Adolesc..

[38] Galambos N.L., Lerner R.M., Steinberg L. (2004). Gender and gender role development in adolescence. Handbook of Adolescent Psychology.

[39] Maccoby E.E. (1990). Gender and relationships: A developmental account. Am. Psychol..

[40] Erdley C.A., Nangle D.W., Newman J.E., Carpenter E.M. (2001). Children’s friendship experiences and psychological adjustment: Theory and research. New Dir. Child Adolesc. Dev..

[41] Rubin K.H., Bukowski W.M., Parker J.G., Eisenberg N., Damon W., Lerner R.M. (2006). Peer Interactions, Relationships, and Groups. Handbook of Child Psychology: Social, Emotional, and Personality Development.

[42] Nangle D.W., Erdley C.A., Newman J.E., Mason C.A., Carpenter E.M. (2003). Popularity, friendship quantity, and friendship quality: Interactive influences on children’s loneliness and depression. J. Clin. Child Adolesc. Psychol..

[43] Gaertner A.E., Fite P.J., Colder C.R. (2010). Parenting and friendship quality as predictors of internalizing and externalizing symptoms in early adolescence. J. Child Fam. Stud..

[44] Kwame S.S., Pamela S.J. (2015). Childhood friendships and psychological difficulties in young adulthood: An 18-year follow-up study. Eur. Child Adolesc. Psychiatry.

[45] Sullivan H. (1953). The Interpersonal Theory of Psychiatry.

[46] Engels C.M.E., Finkenauer C., Meeus W., Deković M. (2001). Parental attachment and adolescents’ emotional adjustment: The associations with social skills and relational competence. J. Couns. Psychol..

[47] Kobak R.R., Sceery A. (1988). Attachment in late adolescence: Working models, affect regulation, and representations of self and others. Child Dev..

[48] Hartup W.W., Stevens N. (1999). Friendships and adaptation across the life span. Curr. Dir. Psychol. Sci..

[49] Campbell A. (1981). The Sense of Well-Being in America.

[50] Cole D.A., Martin J.M., Powers B., Truglio R. (1996). Modeling causal relations between academic and social competence and depression: A multitrait-multimethod longitudinal study of children. J. Abnorm. Psychol..

[51] Dykas M.J., Ziv Y., Cassidy J. (2008). Attachment and peer relations in adolescence. Attach. Hum. Dev..

[52] Thompson R.A., Keller H., Bard K.A. (2018). Twenty-First Century Attachment Theory. The Cultural Nature of Attachment: Contextualizing Relationships and Development.

[53] Thompson R.A., Raikes H.A. (2003). Toward the next quarter-century: Conceptual and methodological challenges for attachment theory. Dev. Psychopathol..

[54] Thompson R.A., Cassidy J., Shaver P.R. (2016). Early Attachment and Later Development: Reframing the Questions. Handbook of Attachment: Theory, Research, and Clinical Applications.

[55] Schneider B.H., Atkinson L., Tardif C. (2001). Child-parent attachment and children’s peer relations: A quantitative review. Dev. Psychol..

[56] Pallini S., Baiocco R., Schneider B.H., Madigan S., Atkinson L. (2014). Early child-parent attachment and peer relations: A meta-analysis of recent research. J. Fam. Psychol..

[57] Recognizing Adolescence. https://apps.who.int/adolescent/second-decade/section2/page1/recognizing-adolescence.html.

[58] Moher D., Liberati A., Tetzlaff J., Altman D.G., Prisma Group (2009). Preferred reporting items for systematic reviews and meta-analyses: The PRISMA statement. PLoS Med..

[59] Carr S. (2009). Adolescent-parent attachment characteristics and quality of youth sport friendship. Psychol. Sport Exerc..

[60] George C., Kaplan N., Main M. (1985). The Adult Attachment Interview.

[61] Armsden G.C., Greenberg M.T. (1987). The Inventory of Parent and Peer Attachment: Individual differences and their relationship to psychological well-being in adolescence. J. Youth Adolesc..

[62] Hazan C., Shaver P. (1987). Romantic love conceptualized as an Attachment Process. J. Pers. Soc. Psychol..

[63] Bartholomew K., Horowitz L.M. (1991). Attachment styles among young adults: A test of a fourcategory model. J. Pers. Soc. Psychol..

[64] Allen J.P., Porter M., McFarland C., McElhaney K.B., Marsh P. (2007). The relation of attachment security to adolescents’ paternal and peer relationships, depression, and externalizing behavior. Child Dev..

[65] Harter S. (1985). Manual for the Self-Perception Profile for Children.

[66] Furman W., Buhrmester D. (1985). Children’s perceptions of the personal relationships in their social networks. Dev. Psychol..

[67] Bukowski W.M., Hoza B., Boivin M. (1994). Measuring Friendship Quality During Pre- and Early Adolescence: The Development and Psychometric Properties of the Friendship Qualities Scale. J. Soc. Pers. Relatsh..

[68] Sharabany R. (1994). Intimate Friendship Scale: Conceptual underpinnings, psychometric properties and construct validity. J. Soc. Pers. Relatsh..

[69] Harter S. (1988). Manual for the Self-Perception Profile for Adolescents.

[70] Weimer B.L., Kerns K.A., Oldenburg C.M. (2004). Adolescents’ interactions with a best friend: Associations with attachment style. J. Exp. Child Psychol..

[71] Rosenberg M. (1965). Society and the Adolescent Self-Image.

[72] Kandel D.B., Davies M. (1982). Epidemiology of depressive mood in adolescents: An empirical study. Arch. Gen. Psychiatry.

[73] Wright P.H., Duck S., Perlman D. (1985). The Acquaintance Description Form. Understanding Personal Relationships: An Interdisciplinary Approach.

[74] Seiffge-Krenke I. (1987). Eine aktualisierte deutschsprachige Form des Offer Self-Image Questionnaire. Z. Differ. Diagn. Psychol..

[75] Kovacs M., Beck A.T., Schulterbrandt J.G., Raskin A. (1977). An empirical approach toward a definition of childhood depression. Depression in Childhood: Diagnosis, Treament and Conceptual Models.

[76] Achenbach T.M., Edelbrock C. (1991). Child behavior checklist. Burlington.

[77] Dziobek I., Fleck S., Kalbe E., Rogers K., Hassenstab J., Brand M., Convit A. (2006). Introducing MASC: A movie for the assessment of social cognition. J. Autism Dev. Disord..

[78] Goldberg L.R., Johnson J.A., Eber H.W., Hogan R., Ashton M.C., Cloninger C.R., Gough H.G. (2006). The international personality item pool and the future of public-domain personality measures. J. Res. Pers..

[79] Mikulincer M., Selinger M. (2001). The interplay between attachment and affiliation systems in adolescents’ same-sex friendships: The role of attachment style. J. Soc. Pers. Relatsh..

[80] Markiewicz D., Doyle A.B., Brendgen M. (2001). The quality of adolescents’ friendships: Associations with mothers’ interpersonal relationships, attachments to parents and friends, and prosocial behaviors. J. Adolesc..

[81] Sánchez-Queija I., Oliva A. (2003). Vínculos de apego con los padres y relaciones con los iguales durante la adolescencia. Rev. Psicol. Soc..

[82] Zimmermann P. (2004). Attachment representations and characteristics of friendship relations during adolescence. J. Exp. Child Psychol..

[83] Sánchez-Queija I., Oliva A. (2015). A longitudinal view of peer-friendship relations and their association with parental attachment bonds. Int. J. Psychol. Psychol. Ther..

[84] Loeb E.L., Stern J.A., Costello M.A., Allen J.P. (2020). With (out) a little help from my friends: Insecure attachment in adolescence, support-seeking, and adult negativity and hostility. Attach. Hum. Dev..

[85] Saferstein J.A., Neimeyer G.J., Hagans C.L. (2005). Attachment as a predictor of friendship qualities in college youth. Soc. Behav. Pers..

[86] Boling M.W., Barry C.M., Kotchick B.A., Lowry J. (2011). Relations among early adolescents’ parent-adolescent attachment, perceived social competence, and friendship quality. Psychol. Rep..

[87] Feeney B.C., Cassidy J., Ramos-Marcuse F. (2008). The generalization of attachment representations to new social situations: Predicting behavior during initial interactions with strangers. J. Pers. Soc. Psychol..

[88] Bauminger N., Finzi-Dottan R., Chason S., Har-Even D. (2008). Intimacy in adolescent friendship: The roles of attachment, coherence, and self-disclosure. J. Soc. Pers. Relatsh..

[89] Shomaker L.B., Furman W. (2009). Parent—Adolescent relationship qualities, internal working models, and attachment styles as predictors of adolescents’ interactions with friends. J. Soc. Pers. Relatsh..

[90] Venta A., Sharp C. (2015). Mentalizing mediates the relation between attachment and peer problems among inpatient adolescents. J. Infant Child Adolesc. Psychother..

[91] Chow C.M., Ruhl H., Buhrmester D. (2016). Reciprocal associations between friendship attachment and relational experiences in adolescence. J. Soc. Pers. Relatsh..

[92] Wong T.K., Konishi C., Cho S.B. (2020). Paternal and maternal attachment: A multifaceted perspective on adolescents’ friendship. J. Child Fam. Stud..

[93] Arrindell W.A., De Groot P.M., Walburg J.A. (1984). The Scale for Interpersonal Behaviour (SIG), Test Manual, Part 1.

[94] Bijstra J.O., Jackson S., Bosma H.A. (1995). Social skills and psycho-social functioning in early adolescence: A three-year follow-up. Int. J. Adolesc. Med..

[95] Wright P.H. (1984). Self-Referent Motivation and the Intrinsic Quality of Friendship. J. Soc. Pers. Relatsh..

[96] Statistics Canada (1995). National Longitudinal Survey of Children: Survey Instruments for 1994–95, Data Collection, Cycle 1.

[97] Veroff V. (1996). An Integration of Friendship and Social Support: Relationships with Adjustment in College Students. Ph.D. Thesis.

[98] Parker G., Tupling H., Brown L.B. (1979). A parental bonding instrument. Br. J. Med. Psychol..

[99] Kobak R.R. (1993). The Adult Attachment Interview Q-Sort.

[100] Zimmermann P. (1992). Interviewmethoden Zur Erfassung von Freundschafts und Gleichaltrigenbeziehungen im Jugendalter [The Friendship and Peer Relations Interview for Adolescence.

[101] Kobak R.R., Cole H., Ferenz-Gillies R., Fleming W., Gamble W. (1993). Attachment and emotion regulation during mother-teen problem-solving: A control theory analysis. Child Dev..

[102] Straus M.A. (1979). Measuring intrafamily conflict and aggression: The Conflict Tactics Scale (CTS). J. Marriage Fam..

[103] Allen J.P., Hall F.D., Insabella G.M., Land D.J., Marsh P.A., Porter M.R. (2001). Supportive Behavior Coding System.

[104] Allen J.P., Porter M.R., McFarland C.F. (2001). The Autonomy and Relatedness Coding System for Peer Interactions.

[105] Coie J.D., Dodge K.A., Coppotelli H. (1982). Dimensions and types of social status: A cross age perspective. Dev. Psychol..

[106] Parkhurst J.T., Asher S.R. (1992). Peer rejection in middle school: Subgroup differences in behavior, loneliness, and interpersonal concerns. Dev. Psychol..

[107] Asher S.R., Dodge K.A. (1986). Identifying children who are rejected by their peers. Dev. Psychol..

[108] Rudolph K.D., Hammen C., Burge D. (1995). Cognitive representations of self, family, and peers in school-age children: Links with social competence and sociometric status. Child Dev..

[109] Collins N.L., Feeney B.C. (2000). A safe haven: An attachment theory perspective on support seeking and caregiving in intimate relationships. J. Pers. Soc. Psychol..

[110] Feeney B.C. (2004). A secure base: Responsive support of goal strivings and exploration in adult intimate relationships. J. Pers. Soc. Psychol..

[111] Brennan K.A., Clark C.L., Shaver P.R., Simpson J.A., Rholes W.S. (1998). Self-report measurement of adult attachment: An integrative overview. Attachment Theory and Close Relationships.

[112] Mikulincer M., Florian V., Tolmacz R. (1990). Attachment styles and fear of personal death: A case study of affect regulation. J. Pers. Soc. Psychol..

[113] Shulman S., Laursen B., Kalman Z., Karpovsky S. (1997). Adolescent intimacy revisited. J. Youth Adolesc..

[114] Margalit M., Ziv O. (1997). Empirical Follow-Up of ORT Center for Learning Skills (Annual Scientific Report for ORT Foundation).

[115] Furman W. (2000). Network of Relationships Inventory: Behavioral Systems Version.

[116] Furman W., Wehner E.A. (1999). The Behavioral Systems Questionnaire—Revised.

[117] Julien D., Markman H., van Widenfelt B. (1986). Interactional Dimensions Coding System Manual.

[118] West W., Rose S.M., Spreng S., Sheldon-Keller A., Adam K. (1998). Adolescent attachment questionnaire: A brief assessment of attachment in adolescence. J. Youth Adolesc..

[119] Weiss M.R., Smith A.L. (1999). Quality of youth sport friendships: Measurement development and validation. J. Sport Exerc. Psychol..

[120] Kenny M.E., Moilanen D.L., Lomax R., Brabeck M.M. (1993). Contributions of parental attachments to view of self and depressive symptoms among early adolescents. J. Early Adolesc..

[121] Berndt T.J., Keefe K. (1995). Friends’ influence on adolescents’ adjustment to school. Child Dev..

[122] Target M., Fonagy P., Shmueli-Goetz Y., Data A., Schneider T. (2007). The Child Attachment Interview (CAI) Protocol.

[123] Achenbach T.M., Rescorla L.A. (2001). Manual for the ASEBA School-Age Forms & Profiles: An Integrated System of Multi-Informant Assessment.

[124] Collins N., Read S. (1990). Adult attachment, working models, and relationship quality in dating couples. J. Pers. Soc. Psychol..

[125] Moretti M.M., McKay S., Holland R. (2000). The Comprehensive Adolescent-Parent Attachment Inventory.

[126] Slaby R., Guerra N. (1988). Cognitive mediators of aggression in adolescent offenders: I. Assessment. Dev. Psychol..

[127] Martinez I., Garcia F., Veiga F., Garcia O.F., Rodrigues Y., Serra E. (2020). Parenting Styles, Internalization of Values and Self-Esteem: A Cross-Cultural Study in Spain, Portugal and Brazil. Int. J. Environ. Res. Public Health.

